# Proteolytic profiling and comparative analyses of active trypsin-like serine peptidases in preimaginal stages of *Culex quinquefasciatus*

**DOI:** 10.1186/1756-3305-5-123

**Published:** 2012-06-20

**Authors:** Andre Borges-Veloso, Leonardo Saboia-Vahia, Patricia Cuervo, Renata C Pires, Constança Britto, Nilma Fernandes, Claudia M d’Avila-Levy, Jose B De Jesus

**Affiliations:** 1 Laboratório de Biologia Molecular e Doenças Endêmicas, Instituto Oswaldo Cruz, FIOCRUZ, Rio de Janeiro, RJ, Brazil; 2 Laboratório de Pesquisa em Leishmaniose, Instituto Oswaldo Cruz, FIOCRUZ, Rio de Janeiro, RJ, Brazil; 3 Laboratório de Biologia de Tripanossomatíl, Instituto Oswaldo Cruz, FIOCRUZ, Rio de Janeiro, RJ, Brazil; 4 Departamento de Engenharia de Biossistemas, Universidade Federal de São João del Rei, São João del Rei, MG, Brazil

**Keywords:** *Culex quinquefasciatus*, Preimaginal stages, Trypsin-like serine peptidases, Peptidases, Zymography

## Abstract

**Background:**

The mosquito * Culex quinquefasciatu s*, a widespread insect in tropical and sub-tropical regions of the world, is a vector of multiple arboviruses and parasites, and is considered an important risk to human and veterinary health. Proteolytic enzymes play crucial roles in the insect physiology including the modulation of embryonic development and food digestion. Therefore, these enzymes represent important targets for the development of new control strategies. This study presents zymographic characterization and comparative analysis of the proteolytic activity found in eggs, larval instars and pupae of *Culex quinquefasciatus*.

**Methods:**

The proteolytic profiles of eggs, larvae and pupa of *Cx. quinquefasciatus* were characterized by SDS-PAGE co-polymerized with 0.1% gelatin, according to the pH, temperature and peptidase inhibitor sensitivity. In addition, the proteolytic activities were characterized in solution using 100 μM of the fluorogenic substrate Z-Phe-Arg-AMC.

**Results:**

Comparison of the proteolytic profiles by substrate-SDS-PAGE from all preimaginal stages of the insect revealed qualitative and quantitative differences in the peptidase expression among eggs, larvae and pupae. Use of specific inhibitors revealed that the proteolytic activity from preimaginal stages is mostly due to trypsin-like serine peptidases that display optimal activity at alkaline pH. In-solution, proteolytic assays of the four larval instars using the fluorogenic substrate Z-Phe-Arg-AMC in the presence or absence of a trypsin-like serine peptidase inhibitor confirmed the results obtained by substrate-SDS-PAGE analysis. The trypsin-like serine peptidases of the four larval instars were functional over a wide range of temperatures, showing activities at 25°C and 65°C, with an optimal activity between 37°C and 50°C.

**Conclusion:**

The combined use of zymography and in-solution assays, as performed in this study, allowed for a more detailed analysis of the repertoire of proteolytic enzymes in preimaginal stages of the insect. Finally, differences in the trypsin-like serine peptidase profile of preimaginal stages were observed, suggesting that such enzymes exert specific functions during the different stages of the life cycle of the insect.

## Background

* Culex quinquefasciatus * is a widespread insect in tropical and sub-tropical regions of the world that is adapted to the urban environment. In addition to disturbing sleep and causing local allergic reactions when bitten, this mosquito represents an important risk to human and veterinary health because it is involved in the transmission of diverse pathogens, including multiple arboviruses, filarial worms and protozoan parasites [1-10]. Due to the absence of effective vaccines against these pathogens, combating the insect vectors has been the only way to control the spread of these diseases [11].

Peptidases are hydrolytic enzymes that cleave peptide bonds in protein chains. Enzymes from the trypsin-like serine peptidase family are ubiquitous in the animal kingdom and are characterized by a catalytic triad composed of serine, histidine and aspartic acid residues [12-14]. In insects, the cleavage of specific proteins by serine peptidases has pivotal roles in oogenesis, immunity, metamorphosis, modulation of embryonic development and nutrition [15-19]. In addition, it has been shown that the expansion of trypsin-like serine peptidase genes in mosquitoes coincides with the development of the hematophagous trait [20]. In fact, serine peptidases are most abundant in the gut of the mosquitoes, where they provide a continuous supply of essential amino acids and energy, from food, for development [17,21]. Furthermore, trypsin-like enzymes secreted in the gut lumen have been implicated in the process of pathogen establishment in several vector insects [22-24].

Given that trypsin-like serine peptidases play essential roles in several physiological processes of mosquitoes, they have been highlighted as potential targets for insect control. The biochemical characterization of these enzymes may provide important clues for the development of new control strategies by either using peptidases as targets or interfering in the production of these enzymes [25-29].

Despite the worldwide impact of * Cx. quinquefasciatus * on public health, little is known about the expression of active peptidases in the immature stages of this species [30]. In this study, we have used zymographic assays to characterize the proteolytic profile from the egg, larval and pupal stages of * Cx. quinquefasciatus *. We demonstrated that eggs, larvae and pupae express a complex profile of peptidases with high activity in alkaline pH and sensitivity to trypsin-like serine peptidase inhibitors. In addition, some peptidases were shown to be unique to each developmental stage, whereas other proteolytic enzymes were expressed in all stages.

## Methods

### Chemicals

All chemicals were purchased from Sigma Chem. Co. (USA), unless otherwise specified. Stock solutions of 1,10-phenanthroline (200 mM) and pepstatin A (1 mg/ml) were prepared in ethanol, whereas *trans*-epoxysuccinyl L-leucylamido-(4-guanidino)butane (E-64, 10 mM) was prepared in water. Phenylmethylsulfonylfluoride (PMSF, 250 mM) was diluted in isopropanol, and Nα-tosyl-L-lysine chloromethyl ketone hydrochloride (TLCK, 100 mM) and N-*p*-tosyl-L-phenylalanine chloromethyl ketone (TPCK, 100 mM) were dissolved in methanol. Peptidase inhibitors were maintained at −20°C.

### Biological materials

Eggs, larvae (L1, L2, L3 and L4) and pupae of *Cx. quinquefasciatus* were obtained from a closed colony derived from insects captured in the Brazilian state of Rio de Janeiro and maintained in the Laboratório de Fisiologia e Controle de Artrópodes Vetores of the Instituto Oswaldo Cruz (Rio de Janeiro). The eggs were collected 2 days after oviposition and immediately lysed. The larvae were kept at 28°C with a photoperiod of 12:12 h (LD).

### Zymographic assays

Eggs, larvae and pupae were washed twice with phosphate-buffered saline (PBS, pH 7.2) and mechanically disrupted in lysis buffer containing 10% glycerol, 0.6% Triton X-100, 100 mM Tris–HCl (pH 6.8) and 150 mM NaCl [31]. The resulting extracts were centrifuged at 14000 × *g* for 30 min at 4°C to remove insoluble material and protein concentration was determined using the Pierce Protein assay, following the manufacturers protocol. Afterwards, samples were resolved as previously described [32]. Briefly, 10 μg of protein from each sample was mixed with SDS-PAGE sample buffer (125 mM Tris (pH 6.8), 4% SDS, 20% glycerol, 0.002% bromophenol blue) and loaded in 12% or 10% polyacrylamide gels co-polymerized with 0.1% porcine gelatin for separation at 4°C with a constant voltage of 110 V. Peptidase activity was detected as previously reported [33] with few modifications. The gels were incubated in the reaction buffer containing 100 mM sodium acetate (at pH 3.5 or 5.5) or 100 mM Tris–HCl (pH 7.5 or 10.0) at 37°C for 0.5, 1, 2 or 4 h for larvae; 0.5, 1, 2, 4, 6, 12 or 24 h for pupa; and 6, 12, 24 or 48 hours for egg homogenates. Bands of gelatin degradation were visualized by staining the gels with 0.2% Coomassie blue R-250 in methanol/acetic acid (40:10) and destaining in 10% acetic acid. The molecular masses of peptidases were estimated by comparison with the mobility of a commercial molecular mass standard (PageRuler™ Protein Ladder, Fermentas). All results are derived from three independent experiments carried out in triplicate.

### Effect of temperature on proteolytic activity

Gels containing larval homogenates were incubated at 10, 25, 37, 50, 65 or 85°C for 2 h in preheated 100 mM Tris–HCl (pH 7.5), and peptidase activity was determined as described above.

### Peptidase inhibition assays

Egg, larvae and pupa homogenates were pre-incubated for 30 min at 37°C with one of the following peptidase inhibitors: 10 μM E-64, 1 mM PMSF, 100 μM TLCK, 100 μM TPCK, 10 μM pepstatin-A or 10 mM 1,10-phenanthroline. After electrophoresis, inhibitors at the same concentration were also added to the reaction buffer, and peptidases were resolved as described above.

### In-solution enzymatic assays

The in-solution determination of peptidase activity from larval homogenates was performed using the fluorogenic substrate Z-carbobenzoxy-L-phenylalanyl-L-arginine-(7-amino-4-methylcoumarin) [Z-Phe-Arg-AMC]. The substrate was prepared at a concentration of 3 mM stock in dimethylsulfoxide (DMSO), from which it was diluted to a 100 μM working solution for each assay. The reactions proceeded by adding 10 μg of proteins from larvae of each instar diluted in 100 mM sodium phosphate buffer (pH 7.5). The fluorescence intensity was measured continuously by spectrophotofluorometry (SpectraMax Gemini XPS, Molecular Devices, CA) using excitation and emission wavelengths of 380 and 460 nm, respectively. All assays were performed at 37°C and pH 7.5. Controls without enzyme or without substrate were also included. All results are derived from three independent experiments carried out in triplicate.

## Results

### Peptidase profile and time course of proteolytic activity from eggs

**Figure 1:**
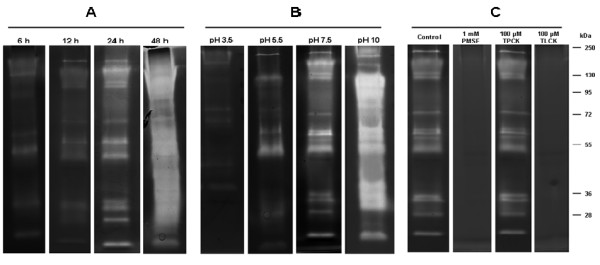
** Time course, influence of pH, and effect of peptidase inhibitors on the proteolytic activity from eggs of* Culex quinquefasciatus*.(A)** For the time course analysis, the proteolytic activity was detected by incubation of SDS-gelatin gels at 37°C in 100 mM Tris–HCl (pH 7.5); **(B)** the influence of pH on the enzyme activity was evaluated after incubation of the gels at 37°C for 24 h in 100 mM sodium acetate (pH 3.5 or 5.5) or in 100 mM Tris–HCl (pH 7.5 or 10.0); **(C)** For evaluating the effect of peptidase inhibitors on the peptidase profile, the samples were pre-incubated for 30 min in the presence of each of the following inhibitors: 1 mM PMSF, 100 μM TPCK or 100 μM TLCK. Then, proteolytic activity was detected after incubation of the gels for 24 h at 37°C in 100 mM Tris–HCl (pH 7.5). The control was processed under the same conditions but in the absence of inhibitors. The numbers on the right of each image indicate the apparent molecular masses of the peptidases, expressed in kDa.

The zymographic profiling from eggs was evaluated between 6 and 48 h of incubation at pH 7.5. The intensity of proteolytic activity increased progressively from 6 to 48 h. However, the complete peptidase profile was better resolved at 24 h because many bands of activity were overlapping at 48 h. Therefore, the 24 h time point was used for all subsequent egg enzymatic assays. Eggs presented a peptidase profile composed of eleven bands ranging from 17 to 200 kDa, approximately (Figure 1A).

### Influence of pH on the proteolytic activity from eggs

To analyze the pH dependence of peptidase activity, the gels were incubated for 24 h in different buffers ranging from pH 3.5 to 10.0. Enzymatic activity was detected at all assayed pH levels. Nevertheless, the intensity of the proteolytic profile at pH 3.5 and 5.5 was drastically reduced when compared with those obtained at pH 7.5 and 10.0 (Figure 1B).

### Enzymatic inhibition assays in egg homogenates

The effect of a number of peptidase inhibitors on proteolytic activity from egg homogenates was determined. TLCK and PMSF strongly inhibited the enzymatic activity whereas TPCK displayed a peptidase profile similar to control (Figure 1C). E-64, pepstatin A, and 1,10-phenanthroline did not affect the enzymatic pattern detected for the egg extracts (data not shown).

### Zymographic profiles and time course of proteolytic activity from larval instars

**Figure 2:**
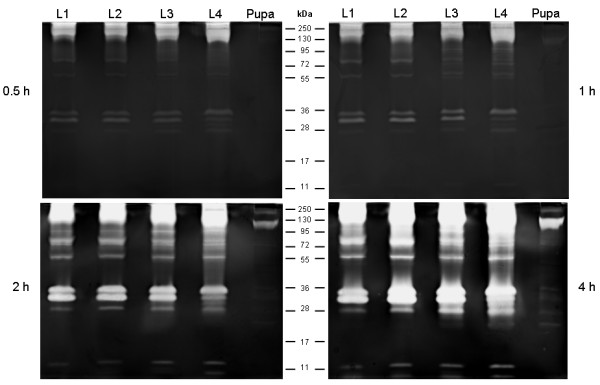
** Time course of proteolytic activity from the four larval instars and pupae of*Culex quinquefasciatus*. ** L1, first instar larva; L2, second instar larva; L3, third instar larva; L4, fourth instar larva. Peptidase activity was detected after incubation of the gels at 37°C in 100 mM Tris–HCl (pH 7.5) for 0.5, 1, 2 or 4 h. The numbers in the middle indicate the apparent molecular masses of the peptidases, expressed in kDa.

The zymographic profiles from L1, L2, L3 and L4 larval instars and pupae were analyzed at 0.5, 1, 2 and 4 h buffered at pH 7.5. The larval peptidase profile was composed of ten or eleven bands ranging from 11 to 130 kDa, approximately. Eight bands of intense activity were detected ranging from 28 to 130 kDa and two bands with low activity were observed at 11 kDa (Figure 2). Although larval enzymatic activity was detected at all experimental conditions (30 min to 4 h), gels allowed to react for 2 h clearly resolved the peptidase composition of all larval instars. Therefore, this time point was used routinely for all subsequent larval enzymatic assays. The experimental conditions used here were adequate for the detection of peptidase activity in all larval instars but did not allow the detection of proteolytic activity in pupae.

### Influence of pH on the proteolytic activity from larval instars

**Figure 3:**
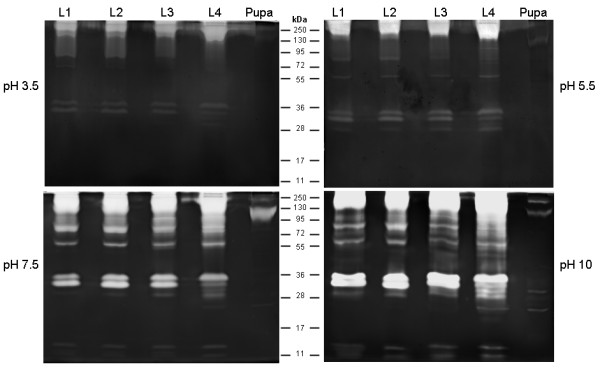
** Influence of pH on the proteolytic profile from the four larval instars and pupae of* Culex quinquefasciatus *. ** L1, first instar larva; L2, second instar larva; L3, third instar larva; L4, fourth instar larva. Enzymatic activity was evaluated after incubation of the gels at 37°C for 2 h in 100 mM sodium acetate (pH 3.5 or 5.5) and 100 mM Tris–HCl (pH 7.5 or 10.0). The numbers in the middle indicate the apparent molecular masses of the peptidases, expressed in kDa.

Enzymatic activity from all larval instars was detected at each pH level tested (Figure 3). Nevertheless, the intensity of the proteolytic profile at pH 7.5 and 10.0 was higher than that observed at pH 3.5 and 5.5. At pH 10.0, some of the proteolytic halos overlapped, precluding the distinction of each band. The experimental conditions used here were adequate for the detection of peptidase activity in all larval instars but were not ideal for detecting proteolytic activity in pupae, suggesting that reaction times and pH conditions had to be determined separately for pupal analysis.

### Enzymatic inhibition assays in larval instar homogenates

**Figure 4:**
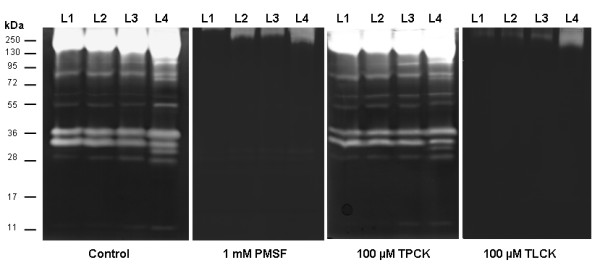
** Effect of peptidase inhibitors on the proteolytic profile from larval instars of* Culex quinquefasciatus *. ** The samples were pre-incubated for 30 min in the presence of each of the following inhibitors: 1 mM PMSF, 100 μM TPCK or 100 μM TLCK. Then, the proteolytic activity was detected after incubation of the gels for 2 h at 37°C in 100 mM Tris–HCl (pH 7.5). The control was processed under the same conditions but in the absence of inhibitors. The numbers on the right indicate the apparent molecular masses of the peptidases, expressed in kDa.

The enzymatic pattern present in the larval homogenates was strongly inhibited by TLCK and PMSF, whereas TPCK displayed a peptidase profile similar to the control (Figure 4). E-64, pepstatin A, and 1,10-phenanthroline did not affect the enzymatic pattern detected for all larval instars (data not shown).

### Effect of temperature on peptidase activity in larval instar homogenates

**Figure 5:**
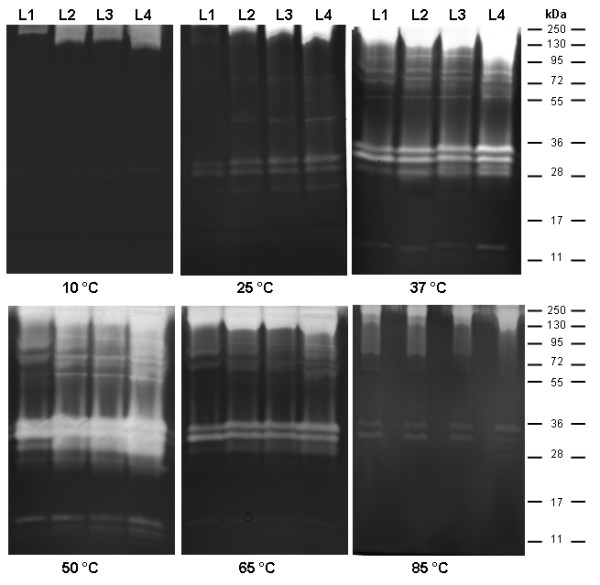
** Effect of temperature on proteolytic activity from larval instars of*Culex quinquefasciatus*. ** Enzyme activity was detected after incubation of the gels for 2 h at 10, 25, 37, 50, 65 or 85°C in 100 mM Tris–HCl (pH 7.5). The numbers on the right indicate approximate molecular masses of the peptidases, expressed in kDa.

Low peptidase activity was observed for all larval instars when samples were incubated at temperatures ranging from 10 to 25°C. Optimal peptidase activity was observed at 37°C, with a strong increase when samples were incubated at 50°C. This activity was reduced at 65 and 85°C (Figure 5).

### Zymographic profiles and time course of proteolytic activity from the pupal stage

**Figure 6:**
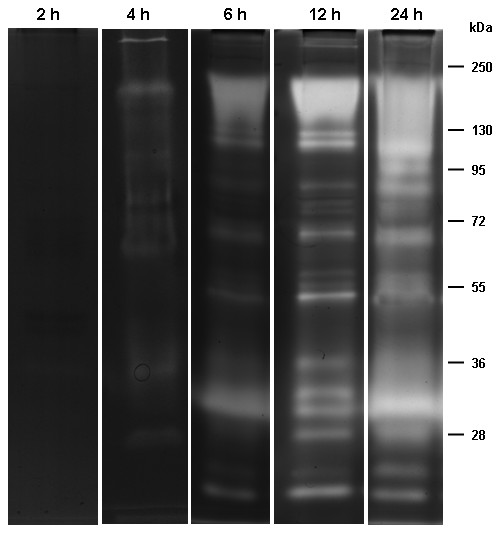
** Time course of proteolytic activity from pupae of* Culex quinquefasciatus *. ** Enzyme activity was detected after incubation for 2, 4, 6, 12 or 24 h at 37°C in 100 mM Tris–HCl (pH 7.5). The numbers on the right indicate the apparent molecular masses of the peptidases, expressed in kDa.

The enzymatic profile from the pupal stage was evaluated using reaction times of 0.5, 1, 2, 4, 6, 12 and 24 h in 100 mM Tris–HCl (pH 7.5). No activity was observed at 0.5, 1 and 2 h, but proteolysis increased progressively from 4 to 24 h (Figures 2 and 6). The enzymatic profile was best resolved at 12 h, allowing the visualization of 15 degradation halos, ranging from approximately 17 to 130 kDa.

### Effect of pH on the proteolytic profile of the pupal stage

**Figure 7:**
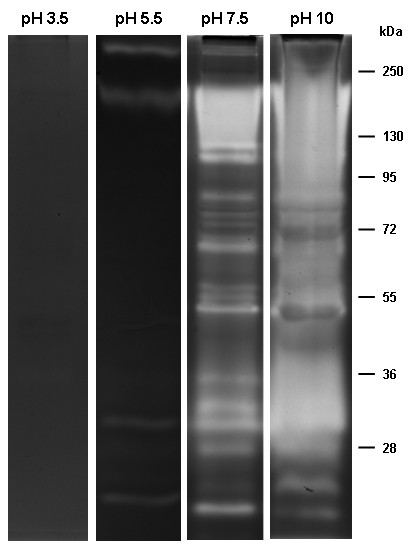
** Influence of pH on the proteolytic profile from pupae of* Culex quinquefasciatus *. ** The proteolytic activity was detected after incubation of gels in 100 mM sodium acetate (pH 3.5 or 5.5) or 100 mM Tris–HCl (pH 7.5 or 10.0), at 37°C for 12 h. The numbers on the right indicate approximate molecular masses of the peptidases, expressed in kDa.

Peptidase activity from pupae was detected at pH levels from 5.5 to 10.0, whereas no proteolytic band was detected at pH 3.5 (Figure 7). Because the bands exhibiting proteolytic activity overlapped at pH 10.0, the pH 7.5 was chosen for the subsequent characterization of pupal peptidases.

### Effect of inhibitors on the proteolytic activity of the pupal stage

**Figure 8:**
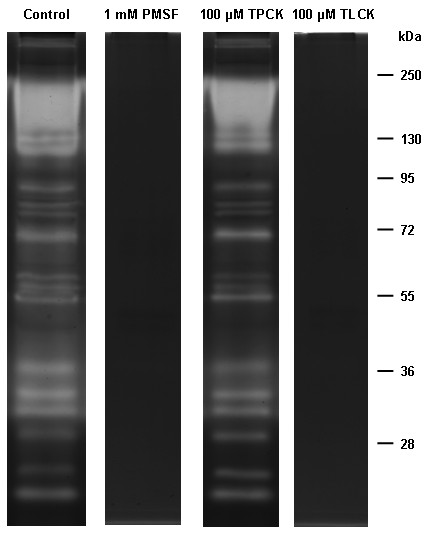
** Effect of the inhibitors PMSF, TPCK and TLCK on the proteolytic profile from pupae of*Culex quinquefasciatus*. ** The proteolytic activity was detected after incubation of the gels for 12 h at 37°C in 100 mM Tris–HCl (pH 7.5). The proteolytic assays were performed in the absence (control) or presence of 1 mM PMSF, 100 μM TPCK or 100 μM TLCK. The numbers on the right indicate approximate molecular masses of the peptidases, expressed in kDa.

The enzymatic profile exhibited by pupal homogenates was strongly inhibited by TLCK and PMSF but not by TPCK (Figure 8). E-64, pepstatin A or 1,10-phenanthroline showed no effect on the peptidase activity of pupae (data not shown).

### In-solution detection of proteolytic activity

**Figure 9:**
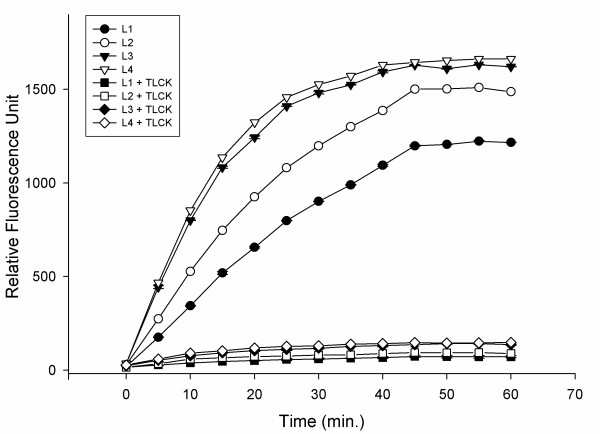
** Detection of proteolytic activity from the four larval instars of* Culex quinquefasciatus *using the fluorogenic substrate Z-Phe-Arg-AMC. ** The proteolytic assays were performed in the absence (control) or presence of 100 μM TLCK in 100 mM sodium phosphate (pH 7.5).

Proteolytic activity from larval instars was determined using the fluorogenic substrate Z-Phe-Arg-AMC in the presence or absence of TLCK, a specific inhibitor of trypsin-like serine peptidases. All larval instar extracts were able to degrade the substrate. Proteolytic activities increased progressively from L1 to L4 with each instar displaying distinct velocities of substrate degradation. All enzymatic activities were strongly inhibited by TLCK (Figure 9).

## Discussion

This study presents zymographic characterization and comparative analysis of proteolytic activity in eggs, larval instars and pupae of * Cx. quinquefasciatus *. The time-course assays demonstrated that the most complete profile of active peptidases were obtained at an incubation time of 24 h for eggs, 2 h for larvae and 12 h for pupae. The proteolytic activity was evaluated both for sensitivity to inhibitors and pH dependence. The use of PMSF, an inhibitor specific for serine peptidases, revealed that serine peptidases are responsible for the proteolytic activity detected for all stages. To determine whether such enzymes were trypsin- or chymotrypsin-like serine peptidases, other class-specific inhibitors were used. All activity bands were strongly inhibited by TLCK, a trypsin inhibitor, indicating that the serine peptidases detected here belong to the trypsin-like family. In addition, in-solution assays performed using the synthetic fluorogenic substrate Z-Phe-Arg-AMC confirmed the results obtained in zymographic analysis using substrate-SDS-PAGE. Serine peptidases form one of the largest families of peptidases in mosquitoes, occurring in different tissues and stages of development [17,34-36]. Trypsin-like and chymotrypsin-like serine enzymes are the dominant digestive peptidases in many larvae and adult insects. Our analyses revealed a much more complex profile of trypsin-like activity in all preimaginal stages of * Cx. quinquefasciatus * when compared with zymographic analyses of other Diptera such as * Ceratitis capitat * and * Lutzomyia longipalpis *, as well as of other insect orders like Lepidoptera (* Achaea janat * and * Heliothis virescens *) and some Coleoptera (* Chrysomya bezziana  Cynaeus angustus  Morimus funereus  Tenebrio molitor * and * Tribolium castaneum *) [24,37-42]. In fact, it has been demonstrated that the trypsin-like serine peptidase family has evolved both in size and in complexity among the mosquitoes. It has been suggested that trypsin-like serine peptidase genes have undergone an expansion in mosquitoes most likely driven by the acquisition of the hematophagous trait. However, the size variation of this gene family is small among mosquito species [20]. Wide proteomic and genomic analyses of *An. gambiae* and *Drosophila melanogaster* identified ~305 and 206 functional trypsin-like genes, respectively [43], whereas, * Cx. quinquefasciatus * and * Aedes aegypti * genomes were shown to contain 403 and 380 trypsin-like serine peptidase genes, respectively [20]. These data indicate that * Ae. aegypti * and * Cx. quinquefasciatus * genomes code for ~46% and ~55% more trypsin-like serine peptidases than *D. melanogaster*[43]. Therefore, the complexity of active serine peptidase expression in preimaginal forms of * Cx. quinquefasciatus * may reflect the expansion of these genes in this mosquito. Previous reports from our group have shown that preimaginal stages of * Ae. aegypti * also exhibit a complex profile of trypsin-like serine peptidases [33], similar to those observed here for * Cx. quinquefasciatus *.

Like most insect serine peptidases, the *Cx. quinquefasciatus* trypsin-like serine peptidases were active over a broad pH range (5.5 to 10.0). Indeed, all activity was strongly detected at pH 10.0 in eggs, larvae and pupae, but the hyperactivity of peptidases at this pH produced overlapping zones and smears in some regions of the gel, causing activity bands to blur and compromising comparative analysis. To avoid this, an optimal pH level of 7.5 allowed for the detection of the complete serine peptidase profile for all analyzed stages. Similarly, larvae of other Diptera such as * Oxysarcodexia thornax, Ae. aegypti * and * Lu. longipalpis * were shown to exhibit serine peptidase activity over a wide range of alkaline pH [32,33,44].

Our results showed that the serine peptidase profile of preimaginal stages of * Cx. quinquefasciatus * is stage-specific, displaying qualitative and quantitative differences in activity detected by zymography. Some trypsin-like serine peptidase activities were found for all stages, suggesting that these enzymes are constitutively expressed during developmental stages and that they exert common functions in all analyzed stages. Comparison of the proteolytic profile of eggs and larvae showed that they share bands migrating between 28 to 36 kDa and 55 to 72 kDa, whereas they differ by a band at 17 kDa that is present in the egg but absent in the larvae, and also by two bands at 11 kDa that are present in the larvae but absent in the eggs. The activity detected for eggs may be supported by the necessity of yolk protein digestion or tissue remodeling during embryological development. In this phase, the hydrolysis of yolk proteins is required to release amino acids and peptides steadily during the first 16 h of pre-embryonic development [30,45]. Serine carboxypeptidases are synthesized in the fat body of *Ae. aegypti*, transported through the hemolymph, and taken up by oocytes [46]. This protease is synthesized as a zymogen and activated within eggs during embryogenesis. Thus, the proteolytic profile detected for the egg homogenates may be composed of activities present in both the pre-embryonic stage and the fully developed larval embryos. In fact, the eggs that were used were two days post oviposition; thus, in this condition, the embryological development had ended, and the embryo was structurally similar to a young L1 instar. Peptidases with specific roles in embryogenesis and hatching have been described in other insects such as *Bombyx mori *[47], * Rhodnius prolixus *[48] and *Lucilia cuprina*[49,50].

The complex proteolytic profile exhibited by the pupal stage also revealed qualitative differences between the profiles of eggs and larvae. It was observed that this stage exhibits one band at approximately 36 kDa that is not present in the other preimaginal stages. We also observed that the pupa displays two bands at approximately 17 kDa, whereas the egg displays only one band in this region. In addition, quantitative differences in the expression of trypsin-like serine peptidase activities were observed during the time-course assays (24 h for eggs, 2 h for larvae and 12 h for pupae). Such differences could be related to the expression of peptidases specifically involved in larval nutrition because the larval instars feed actively whereas pupae and eggs do not. *Cx. quinquefasciatus* larvae can live in water containers with high levels of pollution and microbial fauna, where they feed by filtering various material from the environment. The occurrence of highly active trypsin-like enzymes in larvae when compared with eggs and pupae may be related to the digestion of this complex diet. A large trypsin-like serine peptidase repertoire may help larvae to respond to changes in diet composition or biological inhibitors [17]. This activity then drops after larval-pupa ecdysis and remains at a low level beyond the pupal-adult ecdysis [51]. Trypsin in pupae could be related to proteolysis of the remaining larval tissue during metamorphosis [17]. The differences observed in the proteolytic profiles among the preimaginal stages indicate that the expression of active peptidases is finely regulated. However, the mechanisms involved in such regulation are not yet well understood. The reduction and/or suppression of the expression of peptidases, such as those involved in larval feeding, and the increase and/or induction of the expression of peptidases that have functions in the eggs or pupa themselves, should be under the control of some type of regulation during the life cycle.

It was also observed here that peptidase expression in larvae increases at each larval stage. The quantitative differences in proteolytic activity could be related to the differential expression of these enzymes in the distinct larval instars; this phenomenon has also been observed in other species of Culicidae, specifically in the gut, using methodologies other than zymography [28,52]. The experiments performed with the fluorogenic substrate Z-Phe-Arg-AMC also confirmed this observation. In addition, the zymographic analysis used here facilitated the observation of different bands that appear during larval development, which are not evident by other methods. Comparative analysis of larval instars revealed bands migrating between 25 and 36 kDa at the fourth instar that were not observed in the other larval instars. Therefore, the combined use of zymography and in-solution assays allowed for a more detailed analysis of the repertoire of proteolytic enzymes.

Despite different studies that have reported serine peptidases with high molecular masses [37,39-42], it is expected that monomeric forms of trypsin-like enzymes migrate at 24–35 kDa. Our results, however, showed enzymes with tryptic activity migrating between 55 and 130 kDa. Because the protein samples are not completely denatured or reduced, it is unclear whether the higher molecular weight proteins are actual trypsin polypeptides, protein aggregates or even peptidases bound to some amount of membrane that precipitated at the top of the gels. As mentioned by others, such factors may slow the migration of the peptidases [53]. Membrane-bound trypsin occurs in microvilli and other cell membranes of insect gut [54,55]. Thus, during lysis of preimaginal insect samples, some peptidases may be released with their membrane anchors, which may influence their migration through the gel but not prevent their activity. In spite of such events, it is important to highlight that zymography in substrate-SDS-PAGE is a highly reproducible methodology if protocols are well standardized.

Experiments on the effect of temperature on proteolytic profiles showed that despite low enzymatic activity at 10°C and 25°C, the proteolytic profile of the four larval instars could still be observed. Enzymatic activity increased from 37°C to 50°C, and then decreased at 65°C and 85°C, indicating that trypsin-like serine peptidases of *Cx. quinquefasciatus* larvae support extreme temperature conditions. Nevertheless, incubation above 65°C led to a strong decrease of activity most likely due to the thermal denaturation of the enzymes. Similar results were described for serine peptidases of * Ae. aegypti *[33], *O. ovis*[56] and *T. molitor*[57,58].

## Conclusion

To our knowledge, this is the first zymographic study of trypsin-like serine peptidases in preimaginal stages of *Cx. quinquefasciatus*. We showed that such stages displayed a complex proteolytic profile with multiple bands, which exhibited optimal activity at alkaline pH and over a wide range of temperature. In addition, the comparative approach used here allowed us to detect differences in the expression of active peptidases in the analyzed stages and permitted the assignment of specific trypsin activity to each stage. The presence of multiple bands of activity of trypsin-like serine peptidases in *Cx. quinquefasciatus* may be due to occurrence of multiple coding genes in its genome. Such multiplicity could confer an evolutionary advantage that would allow the insect to cope with a variety of peptidase inhibitors during its life cycle. Understanding of the differential expression of trypsin-like serine peptidase profiles during the *Cx. quinquefasciatus* life cycle is important to determine the role of these enzymes during developmental stages of the insect.

## Competing interests

The authors declare that they have no competing interests.

## Authors’ contributions

JBJ and PC designed the study. ABV, LSV, RCP and NF performed the experimental work. ABV, LSV, PC and JBJ analyzed the data. ABV, PC and JBJ prepared the manuscript with the critical input of CMd’AL and CB. All authors read and approved the final manuscript.
